# A Robust FISH Assay to Detect FGFR2 Translocations in Intrahepatic Cholangiocarcinoma Patients

**DOI:** 10.3390/diagnostics13122088

**Published:** 2023-06-16

**Authors:** Lei Zhang, Hao Zheng, Linyu Xu, Si You, Yuanyuan Shen, Yang Han, Steve Anderson

**Affiliations:** Department of Anatomic Pathology and Histology, Central Laboratory Service, Labcorp Drug Development, 8211 Scicor Dr, Indianapolis, IN 46214, USA; haozheng@whu.edu.cn (H.Z.); linyu.xu@labcorp.com (L.X.); si.you@labcorp.com (S.Y.); yuanyuan.shen@labcorp.com (Y.S.); hanyang2013@aliyun.com (Y.H.)

**Keywords:** FGFR2 translocation, intrahepatic cholangiocarcinoma, break-apart FISH, biallelic translocation

## Abstract

FGFR fusions retaining the FGFR kinase domain are active kinases that are either overexpressed or constitutively activated throughout diverse cancer types. The presence of FGFR translocations enhances tumor cell proliferation and contributes to significant sensitivity to FGFR kinase inhibitors. FGFR2 as an actionable target in intrahepatic cholangiocarcinoma (iCCA) has been tested in many clinical trials. FISH (fluorescence in situ hybridization) and NGS (next-generation sequence) are well-known tools to investigate the translocations of FGFR with multiple or unknown translocation partners. A rapid and robust FISH assay was developed and validated to detect FGFR2 translocations from FFPE specimens in iCCA. The analytical performance of the FISH assay was evaluated for probe localization, probe sensitivity and specificity, and assay precision. Twenty-five archival FFPE specimens from local iCCA patients were tested for FGFR2 translocations. FISH results were correlated with that of NGS on some samples. Biallelic translocations and a novel FGFR2 translocation involving the partner gene, SHROOM3, t(4;10) (q21;q26), were identified in a local iCCA patient.

## 1. Introduction

Intrahepatic Cholangiocarcinoma (iCCA) is the second most common primary hepatic malignancy after hepatocellular carcinoma and is associated with hepatitis virus infection. It accounts for 3% of the malignant tumors of the gastrointestinal system and 15% of primary hepatic malignancies, and has high incidence and mortality in East Asia. The prognosis of patients with cholangiocarcinoma is poor. Surgery is the only potentially curative therapeutic option. However, as most patients present with advanced disease, only approximately one-third of newly diagnosed patients qualify for surgery. For other patients with locally advanced or metastatic disease, the standard-of-care first-line systemic treatment is chemotherapy. The FGFR inhibitor targeting tumors with FGFR rearrangements is becoming a promising therapy and gradually changing the paradigm in the treatment of iCCA. FGFR inhibitors are already recommended for the treatment of patients with FGFR2 rearrangements when diseases have progressed after >1 prior line of systemic therapy, with Pemigatinib approved by both FDA and EMA, and Infigratinib and Futibatinib approved by FDA as well [1,2].

FGFR fusion proteins retaining the FGFR kinase domain are active kinases either overexpressed or constitutively activated throughout diverse cancer types. The presence of FGFR fusions not only enhances tumor cell proliferation but also leads to significant sensitivity to FGFR kinase inhibitors.

FGFR2 as an actionable target in intrahepatic cholangiocarcinoma (iCCA) has been studied in many clinical trials [3,4,5]. Pemigatinib is the first targeted therapeutic agent approved in the United States for cholangiocarcinoma with FGFR2 fusions or rearrangements [6]. After that, Infigratinib was granted accelerated approval by FDA for cholangiocarcinoma with an FGFR2 fusion or rearrangement. Several other approaches, including novel FGFR inhibitors, are being investigated to build upon the success of Pemigatinib and Infigratinib [7]. A rapid and accurate assay to detect FGFR2 translocations, a major type in FGFR2 gene alterations, is critical for patient selection in treatment with FGFR non-selective/selective kinase inhibitors.

Break-apart FISH and NGS are well-known effective tools for investigating translocations with multiple or unknown translocation partners. Ninety FGFR2 translocation partners have been discovered in iCCA thus far, involving inter- and intra-chromosome translocations or intra-chromosome arm translocations [8].

Here, we showed the establishment of a rapid and robust FGFR2 break-apart FISH assay in a CAP/ISO-accredited laboratory and reported a novel FGFR2::SHROOM3 translocation in iCCA patients in China accompanied by a snapshot of the literature-reported FGFR2 rearrangement in iCCA.

## 2. Methods and Materials

### 2.1. Samples

A variety of samples was used for these studies. Normal Metaphase CGH Target Slides (Abbott Molecular Inc., Des Plaines, IL, USA) were used in the probe localization, sensitivity, and specificity assay. Twenty-two FFPE samples from individuals who were known not to have iCCA were tested to establish an FGFR2 FISH database, including 4 normal gallbladder samples, 5 chronic cholecystitis samples, 5 chronic intrahepatic cholecystitis samples, and 8 normal tonsil samples (Fanpu Biotech Inc., Guilin, Guangxi, China). Twenty-five archival FFPE samples from different local patients with iCCA were included in this study (Fanpu Biotech Inc., Guilin, Guangxi, China). Samples used for this analytical validation were remnant tissues that were anonymized and their use for such studies was approved by an ethics board and was found to be acceptable for this use and consistent with ethical and medical standards for clinical research with human samples (Table 1).

### 2.2. FISH Hybridization

**Figure 1:**
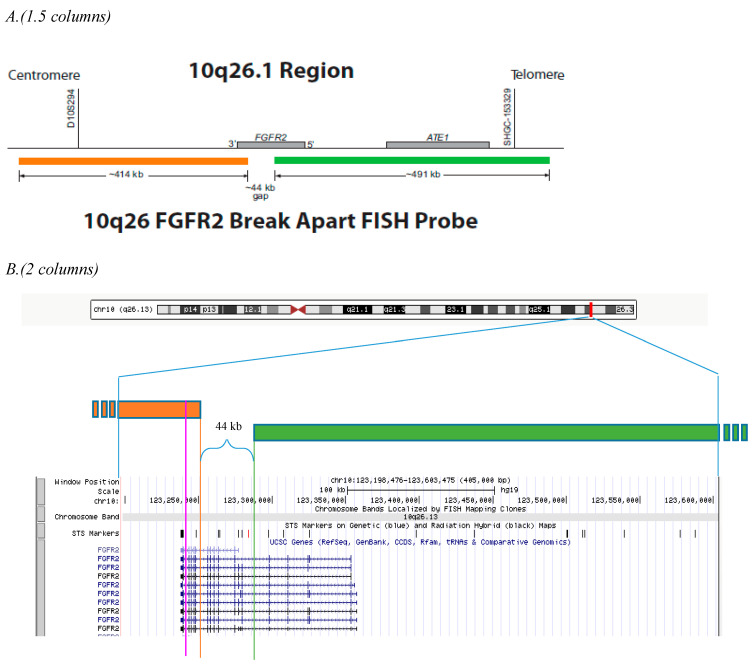
Probe design and configurations. (**A**). The Vysis LSI FGFR2 (Cen) SpectrumOrange probe was positioned centromeric of the FGFR2 gene and was approximately 414 kb in size spanning chr10:122,841,555–123,255,766 on 10q26.12-q26.13 (February 2009, UCSC Genome Browser1). The Vysis LSI FGFR2 (Tel) SpectrumGreen probe was positioned telomeric of the FGFR2 gene and was approximately 491 kb in size spanning chr10:123,300,014–123,791,418 on 10q26.13. (**B**). The orange line indicates the location of Chr10: 123,255,766, the end of the orange probe (Tel), corresponding to the region of the 13th–14th exon of FGFR2. The green line indicates the location of Chr10: 123,300,014, the start of the green probe (Cen), corresponding to the region of the 6th–7th exon of FGFR2. Most FGFR2 breakpoints converge in the region from the last intron distal to the last exon (R). The pink line indicates the breakpoint for the positive case in this study, case 25. As a result of translocation, the green probe will move to the partner chromosomes or gene segments together with a small part of the orange probe and form a big green and a tiny orange break-apart signal (1G1g). Most of the orange probe remains at ch10q26.

FISH was performed on formalin-fixed paraffin-embedded (FFPE) specimens using dual-color break-apart translocation probes specific to the FGFR2 gene (Abbott Molecular, 09N26-060, RUO). See Figure 1 for the probe configuration. The recommended protocol in the probe package insert was referred to. In brief, a Hematoxylin and Eosin (H & E) stained slide was examined by a pathologist to verify tumor content and encircle a representative tumor area for assessment. Then, the encircled tumor area on the H & E slide was used to target the same area on unstained slides by marking the glass slide on the opposite side using an etching tool and then dewaxed and air dried for the next steps. After that, the slides went through the pretreatment (80 ± 1 °C for 25 ± 15 min) and digestion (37 ± 1 °C for 20 ± 10 min) successively (Vysis IntelliFISH Universal FFPE Tissue Pretreatment and Wash Reagents, Abbott, 08N85-005 and Vysis IntelliFISH Protease, Abbott, 08N85-010). The exact time of pretreatment and digestion depended on the pre-analytical conditions of the tissue samples and can be adjusted in the range based on digestion conditions. Then, the denaturation (73 °C for 5 min) was carried out and followed by the hybridization (37 °C for 2 h) in the fast hybridization system (Vysis IntelliFISH Hybridization, Abbott, 08N87-001). After that, the stringent wash was conducted by gently agitating slides in the wash buffer maintained at 73 ± 1 °C for 1 to 3 s (Post-Hybridization Wash Buffer, 08N85-005). Finally, the slides were counterstained (DAPI I Counterstain, Abbott, 06J49-001) and kept at −20 °C until enumeration.

### 2.3. FISH Scoring

Hybridized slides were stored at −20 °C, equilibrated at room temperature, and enumerated by trained readers within 3 weeks. Two readers, working independently and in a blinded fashion, selected different regions within the predetermined tumor area, and each scored 50 tumor cells. In total, 100 tumor cells were scored. A third reader was introduced under the condition that one reader had a positive count at or below the cutoff of 50 nuclei and the other has a count above this value or vice versa. The 3rd reader would count 50 cells and the two scores with the greatest agreement were combined to generate the count for 100 nuclei. According to the database setup, a cell is positive when at least one set of orange and green signals is split apart. Separate signals were defined as signals that do not touch or overlap and were therefore perceived as clearly distinct.

### 2.4. Probe Localization

Metaphase CGH Target Slides (Abbott Molecular Inc., USA) were probed using metaphase protocol. Five metaphase cells were examined through inverted DAPI banding to confirm that the probe correctly hybridized to the chromosomal region to which the probe’s target had been mapped and did not hybridize to other chromosomal regions.

### 2.5. Probe Sensitivity and Specificity

The same metaphase CGH slides were used. The autosomal targets in one hundred cells were examined. The sensitivity of the probes was calculated as the percentage of correct targets detected out of the total number of intended targets. The specificity of the probes was calculated as the percentage of correct targets detected out of the total number of targets detected.

### 2.6. Database, Reference Range

Twenty-two (22) FFPE samples from individuals known not to have cholangiocarcinoma included 8 normal tonsil samples, 4 normal gallbladder samples, and 10 chronic cholecystitis samples. The slides of these samples were analyzed as described above. Cells were scored for the number of different signal patterns. The total number of each signal pattern was tallied. The statistical method that was chosen to calculate the upper limit of the 95% confidence interval for abnormal FISH signal patterns was the mean and the inverse beta function (ACMG guideline recommendation). The mean and beta inverse statistic was performed using the Microsoft Excel program.

### 2.7. NGS

Next-generation sequencing experiments were performed by Precision Scientific (Beijing, China) Co., Ltd. The entire FFPE section was used to extract DNA. DNA libraries were prepared using the KAPA Hyper Prep Kit. Target capture and enrichment were performed by using Precision Scientific proprietary OncoComp enrichment reagents. The 17 intron of FGFR2 gene where most FGFR2 breakpoints converge was included in the capture probe panel. All libraries were sequenced on NextSeq550AR system. Raw sequencing data were mapped to the human reference genome (hg19/GRCh37) using the Burrows–Wheeler Aligner (BWA) version 0.7.17. Sorting, duplicate-read markup, and base-quality score recalibration were performed using the Genome Analysis Toolkit (GATK) version 4.0.12. Further detection of translocations was performed using Precision Scientific in-house software (v0.5.13). Four (4) FISH-negative samples randomly selected from 25 samples and one (1) FISH-positive sample, case 25 were included in the NGS analysis.

## 3. Results

### 3.1. FISH Method Optimization

**Figure 2:**
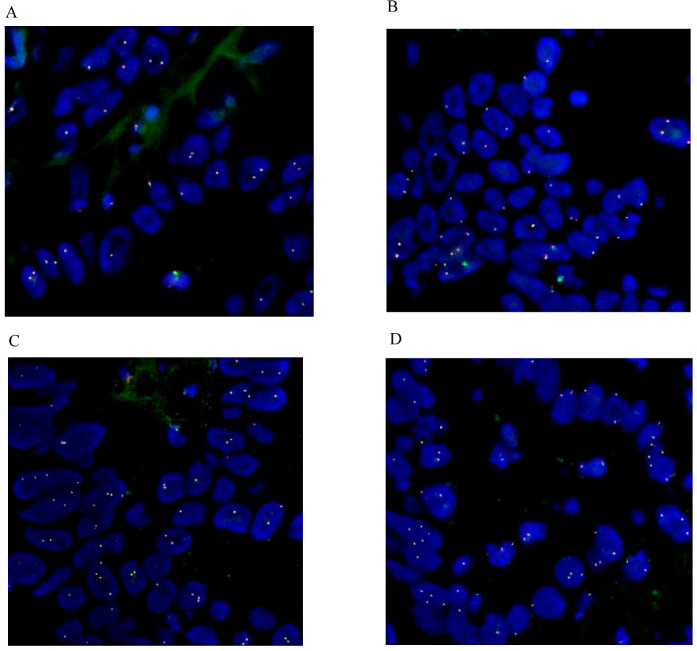
Comparison of two hybridization systems. Two iCCA tissue samples, case 7 and 8, were used in the comparison. The two samples were subjected to FISH analysis (methods and materials) except for the hybridization step. Two hybridization methods were tested separately. Method I, IntelliFISH Hybridization system and hybridization for 2 h at 37 °C. Method II, LSI/WCP Hybridization system and hybridization for 24 h at 37 °C. The signal-to-noise ratio was evaluated. (**A**). Method I, case 7; (**B**). Method II, case 7; (**C**). Method I, case 8; (**D**). Method II, case 8. The shorter hybridization time provided comparable results. (Each of the four images fits one column).

FFPE tissue sections were used to verify the methods and conditions including pretreatment, digestion, denaturation, hybridization, and stringent wash recommended in the probe package insert. Two different hybridization systems were compared, Vysis LSI/WCP Hybridization Buffer (traditional hybridization system) and Vysis IntelliFISH Hybridization Buffer (fast hybridization system). The signal-to-noise ratio was evaluated. A comparable hybridization efficacy was achieved (Figure 2).

### 3.2. Probe Localization, Probe Sensitivity, and Specificity

**Figure 3:**
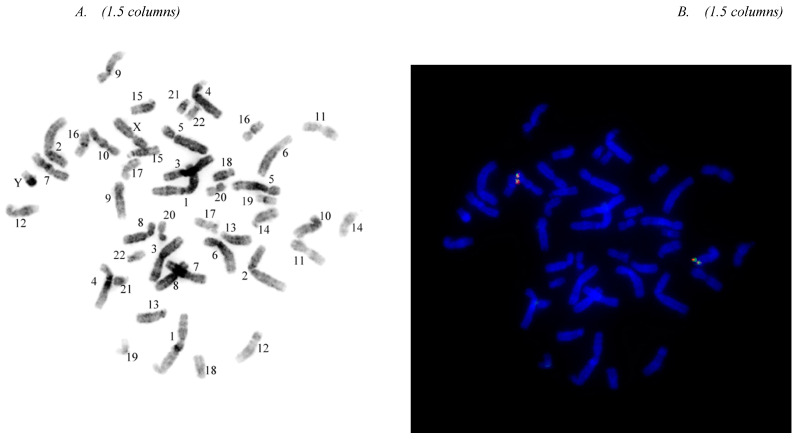
Representative images of probe localization. (**A**). The inversed DAPI banding and chromosome recognition. The signal pattern observed in normal cells without disruption of FGFR2 gene in 10q26.1. Colocalized orange-green hybridization signals, representing the non-rearranged FGFR2 loci, are visible on both homologues of chromosome 10 by metaphase analysis. (Each chromosome has its unique anatomy in terms of chromosome size, location of centromere, arm size, and banding pattern.) (**B**). The same nucleus showed in combined fluorescent channels. The probe signals were mapped onto the intended chromosomal 10 long arms.

Five (5) cells with good metaphase chromosome spreads were examined under inverted DAPI banding mode. Probes were 100% hybridized to the correct site (the long arm of chromosome 10) and no other sites (Figure 3). The same slides were also used to determine the probe sensitivity and specificity. A total of 100 metaphase nuclei (equal to 400 target sites) was examined. Probe sensitivity and sensitivity of 10q26.12-q26.13 (R) were 98.5% and 99.5%, respectively. Probe sensitivity and sensitivity of 10q26.13 (G) were 98.5% and 99.5%, respectively (Table 2).

### 3.3. Database, Reference Range

FISH database is a normal reference range and used for the interpretation of patient samples where acquired mosaic abnormalities exist. The database was constructed based on 22 “normal” FFPE samples. The definition of individual/separate signal was determined as single-color signals with no touch and overlap and perceived as clearly distinct signals. A cell is positive for FGFR2 translocation when at least one set of orange and green signals splits apart. The normal cutoffs were established as 11 for the total count of 50 cells and 19 for 100 cells (Table 3).

### 3.4. FGFR2 Translocation Detection in Twenty-Five (25) iCCA Patients

#### 3.4.1. FISH Results

**Figure 4:**
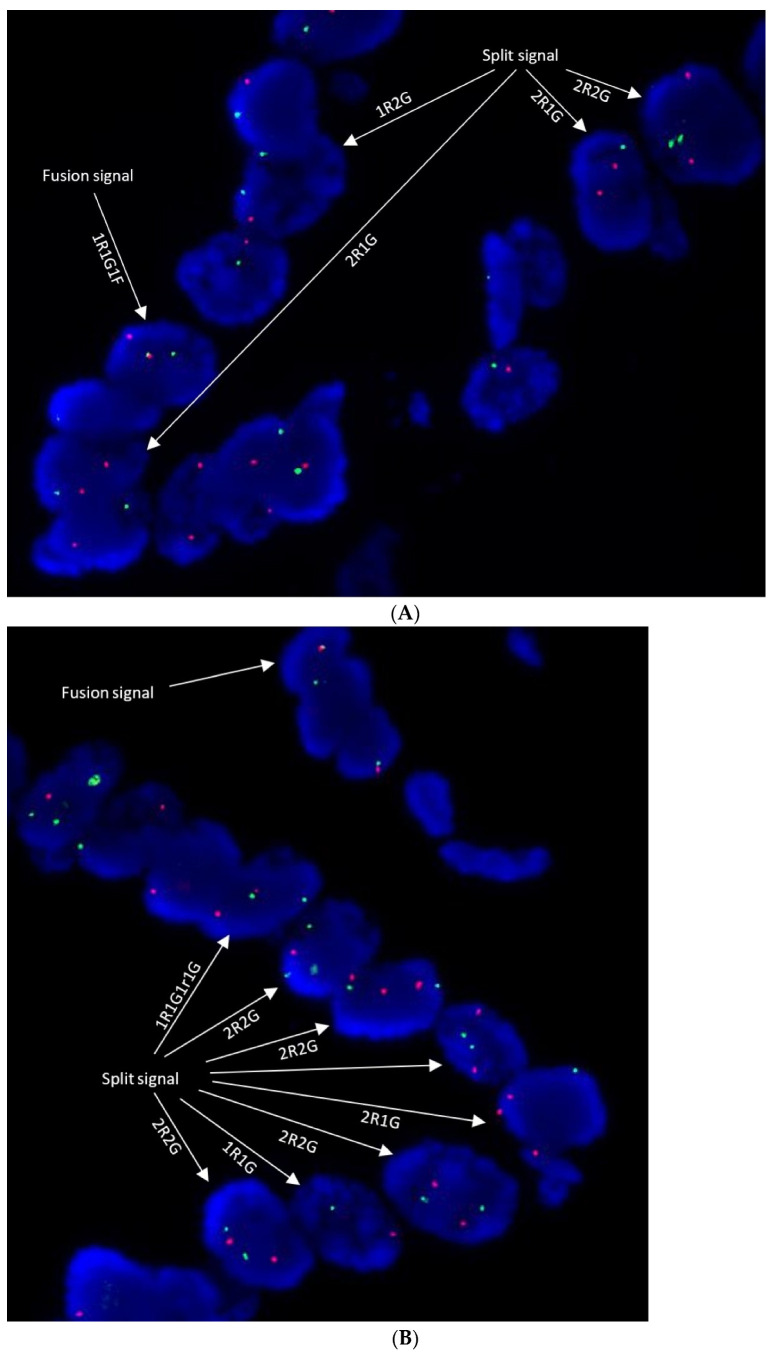
Representative images of the abnormal result (break-apart signals). (**A**,**B**). Different types of break-apart signals were observed in aberrant nuclei including “double split” (2R2G), biallelic translocation, and special 1R1G1r1G signals. R, represents the orange signal. G, represents the green signal. r, represents the small orange signal. (Both (**A**,**B**) fit 1.5 columns).

Twenty-five (25) FFPE iCCA samples were tested using the established FISH method. One of twenty-five (1/25, 4%) samples was positive for FGFR2 translocation, while the remaining 24 samples were negative for FGFR2 translocation. For the positive sample, case 25, the break-apart signal was observed in 98 of 100 (98%) enumerated cells. The observed frequency of FGFR2 translocation in this sample cohort of iCCA was 4% (1/25) which is lower than literature reports (10–15%) [8,9,10], but similar to studies conducted in China iCCA patients (−5%) [11,12]. In addition, the separated signals were seen occurred on both homologous chromosomes (chr10) in many of enumerated nuclei (biallelic translocation), which took the “double split” signal pattern, two sets of split signals (two orange and two green or two orange/green and one green/orange) and zero fusion signals (Figure 4A,B).

#### 3.4.2. NGS Results

Five (5) iCCA samples were subjected to NGS and FISH FGFR2 fusion detection. Consistent results were found in three of five iCCA samples. Two samples detected as negative by FISH were indeterminate by NGS due to sample quality. It indicated that NGS might be more sensitive to FFPE sample pre-analytical conditions (Table 4).

### 3.5. FGFR2::SHROOM3 Translocation

**Figure 5:**
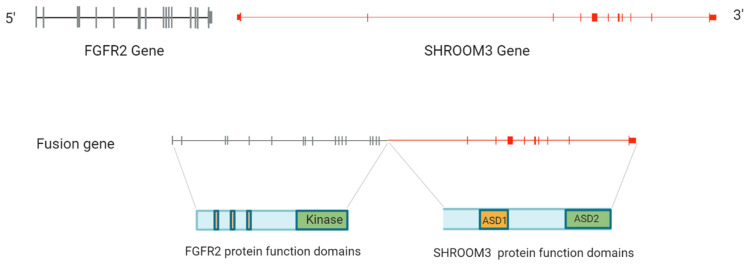
The structure and breakpoints of the fusion genes identified in the study. The upper illustration shows the gene structure of FGFR2 and SHROOM3 genes. The lower illustration shows the fusion gene structure with breakpoint details (in the intron 17 of FGFR2 gene and intron 2 of SHROOM3 gene). (2 columns).

The breakpoints identified in the positive sample by NGS are chr10:123,240,930 and chr4:76,645,542 at a frequency of 4.76%, based on which we constructed the fusion gene structure and breakpoint locations, for the positive sample case 25. The inter-chromosomal translocation between FGFR2 and SHROOM3 gene, t(4;10)(q21;q26), was identified (GRCh38 AR110) in local iCCA patients with a breakpoint within the intron 2 of SHROOM3, which we have not seen reported previously in the literature (Figure 5).

## 4. Discussion

### 4.1. Section Thickness

Fluorescence in situ hybridization, as a molecular cytogenetic method, is used routinely in both cytology and tissue samples. Although a commercial reagent kit provides a general protocol and advice on the range of conditions, a thorough investigation and extensive optimization of the pre-analytic and analytic conditions are critical to produce an acceptable or optimal result, which is especially true when used in FFPE tissue samples. Many factors will cause signal interference, including section thickness and polyploidy of hepatocytes and chromosome territory [13,14,15].

In the beginning of our database setup, we found that the proportion of one fusion (1F) signal pattern was extremely high, 32.60–40.40%. Other polyploidy signal patterns (3F–8F) were also observed. The high proportion of these background noises could skew the database setup and compromise its purpose of investigating the level of background noise of break-apart signal. It had been acknowledged that an age-related increase of polyploidization and nuclear volume is a development feature in hepatocytes in the adult human [14,16,17]. Contamination of hepatocytes in the database setup may cause abnormally high proportion of 1F signal due to nucleus truncation in FFPE slide preparation.

To address the issue of high 1F type signals in the database setup, we first re-enumerate the database samples, chronic intrahepatic cholecystitis samples with particular cautions not to count in hepatocytes to mitigate the interference of the polyploidy and truncated signals. Then, we investigated the potential impact of section thickness on high proportion of 1F signal pattern. Two sections of different thicknesses of the same sample were prepared and analyzed. The results showed that the 1F count of 100 cells in each sample significantly dropped with the increase in section thickness (Table 5). The 1F proportion decreased from the range of 32.60% to 40.40% in sections at 4 μm thickness to 7.69% to 15.09% in sections at 6μm thickness across different types of samples. It indicated that truncation may be the major reason for the observation of a high proportion of 1F. Therefore, the 6μm section thickness was strongly recommended, albeit 4–6 μm was suggested in the probe kit RUO package insert.

### 4.2. Break-Apart Signal Definitions and Its Diverse Signal Patterns

The definition of a separated signal is an important step in the setup of FISH assays employing break-apart probes. Based on it, the occurrence of chromosomal structure anomaly of interest is identified through the observation of signals separation (break-apart) in a nucleus. Three scenarios of signal distribution of break-apart signals can result from translocation events when observed under a fluorescent microscope: (A). where separate orange and green signals take the form of colocalization, touching, or partial overlap due to non-dividing cell nuclear geometry and chromosome steric arrangement. (B). where orange and green signals are determined as clearly distinct signals with no touch and overlap and located less than one signal diameter apart. (C). where orange and green signals are located one signal diameter or more apart. In FGFR2 break-apart FISH assay, a separate signal was defined as signals of different colors located one signal diameter or more apart (C) under a fluorescent microscope. The fusion signals were defined accordingly as colocalized green and orange signals (yellow) or signals of two colors that touch or partially overlap. Adoption of the definitions takes into account the fact that the two probes flanking the known breakpoint cluster region in FGFR2 gene sit only 44 kb apart, which is relatively closer compared to other break-apart probes, for example, ALK (695 kb) [18], ETV1 (530 kb) [19], and FGFR2 BAC probes (162 kb) [9]. This is consistent with what we observed in the database setup: most fusion signals were colocalized green and orange signals exhibiting yellow fusion signals or touching or partially overlapped green and orange signals (Figure 2).

A cell/nucleus was defined as positive when at least one set of orange and green signals split apart or when there is a single green/orange signal in addition to colocalized and/or break-apart signals. In our experiment, multiple types of broken-apart signal patterns were observed with study probes. They were signal patterns, 1R1G1F, 1G1F, 2R2G, 2R1G, and 1R2G (Figure 4A,B). Among them, 1R1G1F or 2R2G were orthodox break-apart signal patterns. Other break-apart signals, such as 1G1F, 2R1G, and 1R2G, may involve additional chromosomal aberrations such as deletions. In addition, we demonstrated the existence of the 1R1G1r1G signal in case 25, which was a break-apart signal pattern specific to the study probe design (Figure 1B and Figure 4B). Theoretically, every break-apart green signal should take the form in aberrant cancer cells where the translocation occurs. However, the 1R1G1r1G signal was only seen in rare cells in case 25. It might be because: 1. The parting-away section of the orange probe only accounts for 3% (12.5 kb/414 kb) of the orange probe. 2. The microscopic observation reduces the 3D nucleic geometry into 2D geometry and the presentation of 1R1G1r1G needs a specific spatial orientation of the chromosomal aberration.

### 4.3. Comparison between Break-Apart FISH and NGS

FISH and NGS are well-known effective tools for investigating translocations with multiple or unknown fusion partners. NGS has advantages in translocation detection by determining breakpoint locations, discovering de novo translocation, and revealing highly complicated rearrangements. However, in the clinical application when it comes to FFPE tissue samples, NGS may have some limitations. DNA-based, hybridization capture-based target enrichment on FFPE material highly depends on the degree of fragmentation. Assessing the integrity of DNA or RNA derived from FFPE material is thus critical to determine which samples are most likely to be successfully prepared for sequencing and producing quality data.

In this study, we adopted the Abbott IntelliFISH hybridization system. It shortened the FISH assay turnaround time by reducing hybridization time from overnight to 2 h. FISH method demonstrated some advantages over NGS in terms of a shorter turnaround time (3–5 d vs. 10–15 d), being less sensitive to FFPE sample pre-analytical conditions, and less consumption of FFPE materials (2–3 slides vs. 4 to 10 slides). Two of five samples subjected to NGS were reported QC failure due to low DNA quality (indeterminate). In the two samples unresolved by NGS, the initial sequencing data showed an average depth in the target region of ~586×. The repeats with doubled DNA input produced only ~783× depth in the target region, far below the target depth of 1000×. It indicated that the extent of DNA fragmentation in the FFPE samples made them unsuitable for NGS fusion analysis. The two samples, however, were analyzed successfully and detected as negative for FGFR2 translocations by the FISH.

Both methods identified the translocation in case 25 in the study. However, the frequencies at which the translocation was detected were markedly different between NGS and FISH with the former being 4.76% and the latter ≥96%. Not conducting a macrodissection of tumor FFPE sections in NGS procedures might be able to explain the much lower frequency of translocation.

FISH technology enables the detection in the context of the tissue and cellular microanatomy, thereby making it a good tool to spatially resolve gene structural or numeric alterations at a chromosomal level. In this study, FGFR2 break-apart FISH assay revealed the “double split” signal pattern (2R2G), FGFR2 biallelic translocation in case 25, which accounted for 60% of positive cells. The FGFR2 monoallelic translocation (1F1G1R or 1F1G) was found in only 4% of positive cells. The remaining positive cell (34%) showed the break-apart signals with a loss of either green or orange signal (1R2G, 2R1G, or 1R1G) (Figure 4A,B), while NGS technology needs first to pulverize specimens and extract nucleic acid before analysis, which makes the characterization a process to destroy and discard the spatial information. From this perspective, NGS is reduced to the detection of genetic variants with somatic mutations in a wild-type normal gene background, which is not intended for the detection of biallelic translocations. From the genetic level, NGS is not yet a phasing technology and cannot resolve haplotypic information.

Biallelic translocations identified by break-apart FISH have been reported in other malignancies. They usually coexist with corresponding monoallelic translocations where a fusion signal remains, indicating the intactness of the other homologous chromosome. A likely explanation for the coexistence of biallelic and monoallelic translocation might be that the aberrant malignant cell population consists of different clones [20,21]. Currently, no research data show if a variation in biological effects, such as gene dose effect, exists between these different translocations. The observed high ratio of non-standard break-apart signals (1R2G, 2R1G, or 1R1G) could be attributed to more complex chromosomal rearrangement events [22] in oncogenesis.

### 4.4. FGFR2::SHROOM3 Translocation

The FGFR2 fusion partner gene, SHROOM3, has been reported previously in iCCA [8,10,11]. In Silverman I et al., 3 of 74 FGFR2-rearranged iCCA cases were identified as translocations with SHROOM3 being the partner gene, of which the breakpoint genomic locations were not released [8]. In Maruki Y. et al., 1 of 21 FGFR2 fusion-positive iCCA cases was found to have SHROOM3 as the fusion partner gene. No breakpoint information was reported in the article [10]. In Zhu Z. et al., 1 of 14 FGFR2 translocation-positive iCCA cases was detected as translocation involving SHROOM2 [11]. The breakpoint was reported in exon 6 of SHROOM, which is different from what we found in the study. In our study, the breakpoint where the head gene segment containing the truncated FGFR2 joined the tail gene segment containing partial SHROOM3 was determined within the intron 2 of SHROOM3 (Figure 5).

Oncogenic mechanisms of FGFR2 rearrangements which give rise to phenotypes of overexpression or constitutive activation were identified as promotor switching or acquisition of oligomerization domains from partner genes, respectively [23]. An instance of the promotor switching is the case of SLC45A3::FGFR2 fusion with FGFR2 at 3′gene (tail gene) position. It contains most of the promoter region of SLC45A3 and only the non-coding region of exon 1 and results in overexpression of FGFR2 in some patients with prostate cancer. Data supporting the acquisition of oligomerization domain in FGFR2 fusions as one of the important mechanisms are accumulating. Different types of oligomerization domains were involved. The coiled coil domain was found in FGFR2::CCDC6 (breast cancer, iCCA), FGFR2::CIT (lung carcinoma), and FGFR2::KIAA1967 (lung squamous cell carcinoma) fusions, all of which exhibited constitutive dimerization [23]. The sterile alpha motif (SAM) domain is another oligomerization domain found in FGFR2::BICC1 fusion in metastatic cholangiocarcinoma. Therefore, FGFR2::BICC1 is another example of ligand-independent dimerization, most likely mediated by the presence of the SAM domain [23]. LIS1-homologous (LisH) domain, a very stable dimerization domain, was found in FGFR2::OFD1 fusion containing a LisH motif and five coiled-coil domains [24]. We interrogated LOGICOIL (http://coiledcoils.chm.bris.ac.uk/LOGICOIL/) (accessed on 4 February 2023) for oligomerization domains in SHROOM3. Both tetramer and antiparallel dimer were suggested. We assumed that the fusion protein encoded by FGFR2::SHROOM3 may acquire the oligomerization capability through SHROOM3, hence enabling malignant cells to come into possession of the oligomerization-induced and ligand-independent constitutive activation of FGFR2 receptor kinase in case 25. Furthermore, studies also showed that the constitutive activation of FGFR2 kinase resulting from FGFR2 rearrangement may be in part contributed by the truncation of FGFR2 in its C-terminal (a universal feature of FGFR2 rearrangements) in the process of forming various kinds of FGFR2 rearrangement aberrations, which leads to the “loss of molecular brake” or escape from microRNA regulation [8,25,26].

**Figure 6:**
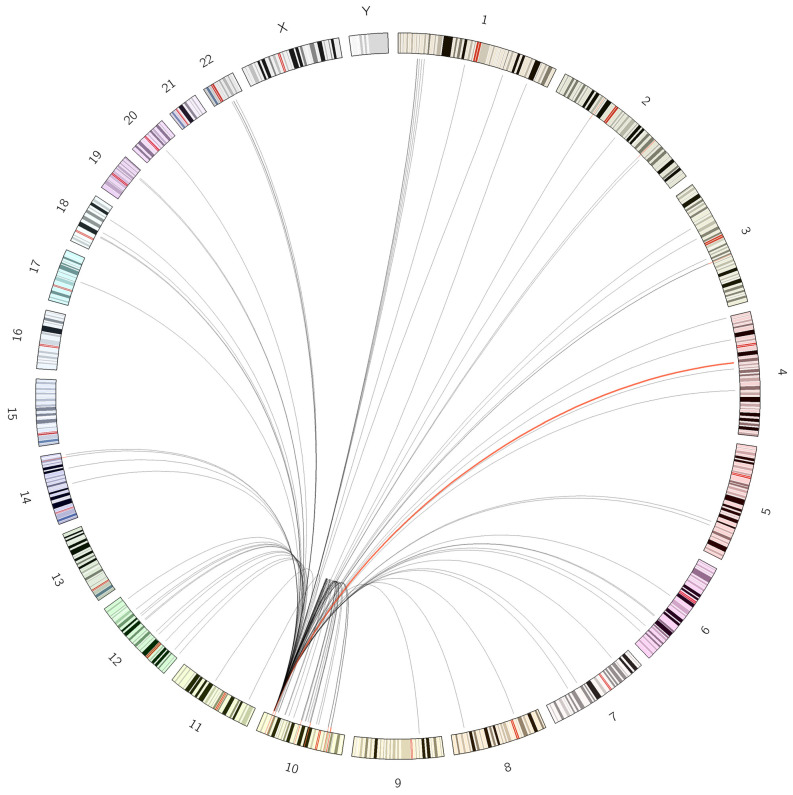
Visualization of FGFR2 partners distribution in genome by Circos ideogram. The link highlighted in red color indicated the translocation, FGFR2::SHROOM3, identified in this study. Highlighted strokes were placed to indicate the 5′ head gene positions in the radial direction immediately adjacent to the inner arc of chromosomal ideogram. For example, on chr10, six locations were highlighted as head gene positions, FGFR2 gene (chr10: 121,593,967–121,598,444), CASC2 gene (chr10: 118,045,862–118,216,096), BICC1 gene (chr10: 58,512,220–58,831,435), VCL gene (chr10: 73,995,193–74,121,363), WAC gene (chr10: 28,532,493–28,623,112), and KIAA1217 gene (chr10: 23,694,727–24,547,848). Other head gene positions were highlighted on chr2, chr3, and ch14, respectively. (2 columns).

When searching previously published data to paint a full picture of SHROOM partner gene in FGFR2 rearrangement, we summarized FGFR2 rearrangement in iCCA in terms of the genomic involvement of its partners based on both the data reported in the literature and an interrogation of the public fusion databases (Table 6 and Figure 6) [8,9,10,11,24,25]. The fusion database, ChimerDB version 4.0, was queried in generating the summarized data, covering both publications (ChimerPub plus) and curated fusion sequences (ChimerSeq plus). To date, 90 partner genes have been identified and reported in FGFR2 rearrangement in iCCA. It contains inter-chromosomal, intra-chromosomal, and intra-chromosomal arm rearrangements and can be categorized into two types, gene-intragenic and gene-intergenic rearrangements. Some reported cases of the latter category were not included in the Circos (Figure 6) due to the lack of information on their genomic locations. Most FGFR2 gene rearrangements in iCCA have FGFR2 as 5′ head gene, with a breakpoint usually found in the region from the last intron distal to the last exon. As a result, the extracellular and transmembrane domains of the FGFR2 protein remain intact, as well as a significant portion of the kinase domain. Only a few of them were reported having FGFF2 as 3′ tail gene, with a breakpoint in the last exon or second last exon [26]. A wide distribution of its partner in genome was revealed, involving all chromosomes except for chr13, chr15, chr16, chr21, chrY, and chX (Figure 6). It is unclear why FGFR2 gene rearrangement occurs with such a profound partner diversity. Possibilities could be that the genetic locus bearing FGFR2 gene is more accessible across a multitude of chromatin conformations based on major ideas from the research of the mechanistic rationale for fusion formation [27,28] and widespread availability of oligomerization domains in human genome [29,30,31].

## 5. Conclusions

FGFR2 as an actionable target in iCCA has been studied in many clinical trials. FGFR2 translocation is a common genetic lesion in iCCA and increasingly used as a biomarker to predict the sensitivity to FGFR kinase inhibitors in iCCA-targeted therapy. Here, a rapid and robust FGFR2 FISH assay was developed and validated for detecting FGFR2 translocations in iCCA from archival FFPE specimens. Compared to NGS, the FISH assay may have potential advantages in clinical applications such as a shorter turnaround time, less consumption of tissue samples, more tolerance to sample pre-analytical conditions, and spatial resolving of translocations. Additionally, a novel FGFR2 translocation involving the SHROOM3 gene (intron 2) was identified in local iCCA patients.

## Figures and Tables

**Table 1:** The demographic information of 25 patients with cholangiocarcinoma.

Case No.	Age	Gender	Location	Histology Type	Stage	TNM_T	TNM_N	TNM_M
1	73	F	Liver	Cholangiocarcinoma	II	T2	N1	M0
2	38	F	Liver	Cholangiocarcinoma	III	T2	N0	M0
3	51	M	Liver	Cholangiocarcinoma	II	T2	N1	M0
4	42	M	Liver	Cholangiocarcinoma	II	T2	N1	M0
5	53	M	Liver	Cholangiocarcinoma	II	T2	N0	M0
6	64	F	Liver	Cholangiocarcinoma	III	T2	N0	M0
7	63	M	Liver	Cholangiocarcinoma	II	T2	N0	M0
8	64	F	Liver	Cholangiocarcinoma	I	T2	N0	M0
9	26	F	Liver	Cholangiocarcinoma	II	T2	N0	M0
10	66	M	Liver	Cholangiocarcinoma	III	T2	N0	M0
11	58	M	Liver	Cholangiocarcinoma	II	T2	N0	M0
12	60	M	Liver	Cholangiocarcinoma	II	T3	N1	M0
13	72	F	Liver	Cholangiocarcinoma	I	T3	N1	M0
14	56	F	Liver	Cholangiocarcinoma	I~II	T2	N1	M0
15	47	F	Liver	Cholangiocarcinoma	II	T2	N0	M0
16	47	M	Liver	Cholangiocarcinoma	II	T2	N0	M0
17	70	M	Liver	Cholangiocarcinoma	II	T2	N0	M0
18	47	M	Liver	Cholangiocarcinoma	III	T2	N0	M0
19	29	F	Liver	Cholangiocarcinoma	II	T2	N0	M0
20	57	F	Liver	Cholangiocarcinoma	II	T2	N1	M0
21	66	F	Liver	Cholangiocarcinoma	III	T2	N1	M0
22	64	F	Liver	Cholangiocarcinoma	I	T1	N0	M0
23	51	F	Liver	Cholangiocarcinoma	II~III	T2	N0	M0
24	61	F	Liver	Cholangiocarcinoma	I~II	T1	N0	M0
25	33	F	Liver	Cholangiocarcinoma	I	T2	N1	M0

**Table 2:** Probe sensitivity and specificity results.

Configurations	Normal	Other Normal Signal Patterns	Break-Apart
Signal patterns	2F	1F	1R1G1F
100 target cells (400 targets)	97	2	1
		Probe sensitivity	Probe specificity
10q26.12-q26.13 (R) *		98.5% (197/200)	99.5% (197/198)
10q26.13 (G) ^∥^		98.5% (197/200)	99.5% (197/198)

*, the probe targeting the chromosome region 10q26.12-q26.13 and labeled with SpectrumOrange. ∥, the probe targeting the chromosome region 10q26.13 and labeled with SpectrumGreen.

**Table 3:** Cutoffs established for the break-apart FGFR2 FISH assay.

Signal Patterns	BA *	1F ^∥^	3F	4F	5F
Cutoffs (number of cells with the signal pattern in 100 cells enumerated)	7	25	11	10	5
Signal patterns	BA	1F	3F	4F	5F
Cutoffs (number of cells with the signal pattern in 50 cells enumerated)	5	14	7	7	4

* BA represents the break-apart signal patterns. F **^∥^** represents fusion signal patterns.

**Table 4:** FISH and NGS results.

Case No.	FISH	NGS
10	Negative	Indeterminate
2	Negative	Negative
1	Negative	Negative
25	Positive	Positive
24	Negative	Indeterminate

**Table 5:** Impact of section thickness on the level of background noise 1F ^∥^.

Tissue Type	1F Ratio at 4 μm, %	1F Ratio at 6 μm, %
Normal liver	40.00	9.23
Normal gallbladder	40.40	18.03
Hepatitis	32.60	7.69
Chronic cholecystitis	38.80	15.09
Chronic intrahepatic cholecystitis	37.20	13.24
Average	37.80	12.66

^∥^, F represents fusion signal patterns.

**Table 6:** Summary of FGFR2 translocations partners in iCCA.

5′ HEAD Genes	3′ TAIL Partners	FGFR2 Partner Chromosomal Locations
FGFR2	BICC1	chr10
	KIAA1217	chr10
	AHCYL1	chr1
	CCDC6	chr10
	TACC2	chr10
	SHROOM3	chr4
	AFF4	chr5
	ARHGAP24	chr4
	CCDC170	chr6
	FILIP1	chr6
	MACF1	chr1
	NOL4	chr18
	NRAP	chr10
	PAWR	chr12
	SLMAP	chr3
	TACC1	chr8
	TRIM8	chr10
	ACLY	chr17
	ARHGAP22	chr10
	ATAD2	chr8
	ATF2	chr2
	BICD1	chr12
	CCDC158	chr4
	CDC42BPB	chr14
	CEP128	chr14
	COL16A1	chr1
	CTNNA3	chr10
	DBP	chr19
	DNAJC12	chr10
	EEA1	chr12
	EIF4ENIF1 (intergenic)	chr22
	ERC1	chr12
	GAB2	chr11
	GOPC	chr6
	INSC	chr11
	KCTD1	chr18
	SHTN1/KIAA1598	chr10
	MATR3	chr5
	MCU	chr10
	NEDD4L	chr18
	NRBF2	chr10
	PAH	chr12
	POC1B	chr12
	PXN	chr12
	RABGAP1L	chr1
	RASSF4	chr10
	ROBO2	chr3
	RPAP3	chr12
	SFI1	chr22
	SOGA1	chr20
	SPICE1	chr3
	STRN4	chr19
	TBC1D1	chr4
	TCTN3	chr10
	TFEC	chr7
	TTC28	chr22
	TXLNB	chr6
	USH2A	chr1
	VCL (intergenic)	chr10
	WAC	chr10
	WDHD1	chr14
	ZMYM4	chr1
	ZNF521	chr18
	MGEA5/OGA	chr10
	TACC3	chr4
	CREB5	chr7
	CCDC186/C10orf118	chr10
	FRK	chr6
	PPFIA2	chr12
	TLN1	chr9
	BAIAP2L1 (intergenic)	chr7
	TNIP3	chr4
	LINC00863	chr10
	CBX5 (intergenic)	chr12
	CFAP57	chr1
	PRDX3	chr10
	GOLGB1	chr3
	ETV6	chr12
	TFCP2L1	chr2
	TXLNA	chr1
	RBFOX2	chr22
BICC1	FGFR2	chr10
VCL		chr10
*CASC2*		chr10
WAC		chr10
***ERICH2-DT***		chr2
KIAA1217		chr10
GOLGB1		chr3
***GMCL1***		chr2
***SETD3***		chr14

Notes: Gene name highlighted in bold and italic fonts were partners which only showed in translocations taking FGFR2 as 3′ tail gene.

## Data Availability

No new data were created or analyzed in this study. Data sharing is not applicable to this article.
